# A cross-sectional quantitative analysis of production and requirements of medical oxygen during the COVID-19 pandemic in Nepal

**DOI:** 10.1136/bmjopen-2024-091189

**Published:** 2025-09-11

**Authors:** Samir Kumar Adhikari, Yeshoda Aryal, Anant Nepal, Melissa Beth Bingham, Subash Neupane, Ashok Basnet, Amit Kumar Singh, Bigyan Prajapati, Deepesh Sthapit, Gaurav Devkota, Samriddha Rana

**Affiliations:** 1 Government of Nepal Ministry of Health and Population, Kathmandu, Nepal; 2 World Health Organization, Kathmandu, Nepal

**Keywords:** COVID-19, PUBLIC HEALTH, Emergency Service, Hospital

## Abstract

**Abstract:**

**Objectives:**

Medical oxygen supplementation is essential for treating severe illnesses and plays a critical role in managing life-threatening conditions, especially during the period of increased demand, such as the delta wave of COVID-19. The study aims to evaluate oxygen requirements and production to support effective capacity planning for future health crises.

**Design and setting:**

Cross-sectional quantitative study. Data collection was carried out between 15 March and 19 December 2021.

**Main outcome measures:**

The study used secondary data from Nepal’s Health Emergency Operation Centre. Regarding medical oxygen production, calculations included oxygen generated from both hospital-based oxygen plants and private companies, using their highest capacities for comparison. These production capacities were then assessed using three levels of efficiency (100%, 80% and 50%), revealing significant gaps when compared against the oxygen requirements of hospitalised COVID-19 patients, as guided by WHO recommendations. The results were communicated in terms of J-size cylinders, alongside average daily COVID-19 hospitalizations. Data was inputted and analysed using Microsoft Excel and presented in numbers and percentage.

**Results:**

The country’s oxygen demand relies largely on the production from private enterprises, with meeting approximately 85.2% of the total requirement. Optimal production ensures that national oxygen needs will be met. The analysis highlighted that at 80% operational efficiency, 90.8% of the hospital’s requirements could be fulfilled. However, if operational efficiency drops to 50%, the fulfilment rate diminishes to 56.7%. The differences in requirement and production of oxygen are consistent across the provinces; however, a huge disparity was notable in Karnali and Sudurpaschim.

**Conclusion:**

Continuous assessment of production capacities in both hospital and private enterprises producing oxygen is necessary to plan and address the gaps.

**STRENGTHS AND LIMITATIONS OF THIS STUDY**

- This is the first study to compare the production and demand for medical oxygen in Nepal during the pandemic, encompassing both public and private hospitals.
- The study employs a comprehensive methodological approach, calculating and analysing daily hospitalised cases, which are then graphically presented to illustrate trends at both the national level and across all seven provinces.
- The study focused on hospitalised COVID-19 cases without accounting for variant types or disease severity, limiting generalisability and likely underestimating oxygen needs, as many severe cases were not hospitalised due to resource or access limitations.
- Oxygen production data during the delta wave were collected via phone interviews, introducing potential recall or reporting bias.
- The study excluded oxygen concentrators and imported liquid oxygen – both of which, while used to a limited extent, contributed to the overall supply.

## Introduction

 Coronavirus disease (COVID-19) is an infectious disease caused by the SARS-CoV-2 virus. Emerging from Wuhan, China, in December 2019, it swiftly spread worldwide, becoming a matter of international concern.^1^ In response, numerous countries implemented containment measures, strengthened hospital capacities and expedited vaccination campaigns. The pandemic resulted in millions of deaths globally^2^ and significantly disrupted lives across the globe.

In response to the COVID-19 pandemic, Nepal's Ministry of Health and Population (MoHP) activated the Incident Command System (ICS) on 26 February 2020, following the classification of COVID-19 as a Public Health Emergency of International Concern (PHEIC) in January 2020.^3^ As COVID-19 cases spread beyond China, it was officially declared a pandemic on 11 March 2020.^4^ The Health Emergency Operation Centre (HEOC), MoHP, Nepal began issuing daily COVID-19 situation reports, using data gathered from Provincial Health Emergency Operation Centers (PHEOCs), hubs and satellite hospitals. As the demand for oxygen surged and proved insufficient to meet requirements during the period of the Delta variant,^5^ the HEOC also started tracking medical oxygen production data to facilitate effective management of COVID-19 cases.

Medical oxygen is crucial for treating patients with acute illnesses, particularly in intensive care units (ICUs) where about half of the patients require it.^6^ It’s listed as an essential medicine by the WHO^7^ for both acute and chronic conditions causing low oxygen levels.^8^ This importance has been highlighted not only during the COVID-19 pandemic^9^ but also in other emergencies,^10^ including severe acute respiratory syndrome.^11^ Despite its essential status, some countries didn't recognise medical oxygen as a public health concern, but the COVID-19 pandemic shifted this perspective.^12^ However, managing the medical oxygen system is complex due to various production sources, storae methods and transportation, requiring expertise from both medical and engineering fields,^13^ which poses challenges for effective management.

During the Delta wave in Nepal virus transmission significantly increased^14^ in line with global trends.^15^ The increased transmissibility of the Delta variant led to more hospitalisationsions, surpassing previous waves. Consequently, there was a greater need for oxygen to treat severe cases.^16^ Despite operating at lower capacity before the pandemic, Nepal had numerous commercial oxygen producers employing air separation units (ASU) or cryogenic separation. These producers ramped up production and directed more oxygen to healthcare, managing to address the escalated demand.

Likewise, on a relatively lower scale, the private sector also commenced supplying liquid oxygen to hospitals from this point in time. However, there has been an absolute dependency on India for imported oxygen.^17^ This dependency poses a risk of supply disruptions. Although smaller in number, some hospitals in Nepal also operated in-house pressure swing adsorption (PSA) oxygen plants. PSA systems separate oxygen from air using adsorption materials, typically achieving approximately 90–95% purity at moderate cost. In contrast, cryogenic separation involves liquefaction and distillation, enabling oxygen purity greater than 99%, but with higher capital and operational costs.^18^

The 76th World Health Assembly, held in May 2023, adopted a resolution (WHA76.3) emphasising increased access to medical oxygen and the need for surge capacity in emergencies, integrating it into health systems for universal health coverage.^19^ Despite numerous studies on oxygen in various countries, documentation of oxygen response in Southeast Asian countries like Nepal during the COVID-19 pandemic is limited.^20^ Existing studies in Nepal focus on oxygen supply-demand gaps in rural areas^5^ and oxygen availability in COVID-19 treatment facilities, considering sources from companies and local production.^21^ However, these studies lack a comprehensive national perspective and fail to address the country’s requirements. This study aims to analyse oxygen requirement and production during the COVID-19 pandemic’s delta variant period, contributing to effective surge capacity planning and future oxygen needs preparation.

## Methods

The study employed a cross-sectional quantitative research design using secondary data obtained from the HEOC in Nepal. In response to the global COVID-19 pandemic, which was officially declared on 11 March 2020,^22^ the HEOC initiated the publication of daily COVID-19 situation reports, gathering data from hospitals. During the surge of the delta variant, a significant gaps between the demand and supply of oxygen were observed, with a notable increase in oxygen requirements compared to previous variant phases.^23^ Consequently, the HEOC began collecting information on oxygen demand and supplies.

Data pertaining to COVID-19 cases and the oxygen situation were collected and analysed specifically during the timeframe of the delta wave. While the period encompassing the delta wave was considered to be from 15 March 2021^24^ to 19 December 2021 (Note in box 1), the HEOC commenced updating reports from 12 April 2021. Hence, the analysis only includes data from 12 April 2021 and 19 December 2021.

Box 1 **Period of Delta and Omicron detection**

Delta variant detection and subsequent continuous increase in the cases from 15 March 2021, lowest number reported on 19 December 2021. Omicron variant detection and subsequent continuous increase in cases from 20 December 2021.

The information underwent analysis and was subsequently showcased using three distinct approaches:

1. An assessment was conducted on COVID-19 cases who were hospitalised between 12 April 2021 and 19 December 2021. This method involved the calculation and analysis of daily hospitalised cases and then the trends for each day were then graphically depicted for both the entire nation and the seven individual provinces.
2. To facilitate a comparison of medical oxygen production capacities between hospitals and private companies, data were gathered via telephone conversations with COVID-19-dedicated hospitals and private oxygen manufacturers in May 2021. This data pertained to the highest capacity figures. The production calculations included oxygen generated from hospital-based PSA oxygen plants, liquid oxygen and oxygen production by private companies.
3. In order to compare oxygen requirement and production capacities, the focus was on the surge period during the delta wave, spanning from 6 May 2021 to 5 June 2021 (details in box 2). This analysis encompassed the calculation of hospitalisations across different hospital units and analysed it in average per day. The requirement for the oxygen was then calculated using recommended standard flow rates per patients hospitalised in different units established by WHO. As per WHO guidelines, the average input flow rates were designated as 10 L/min for severe patients and 30 L/min for critical patients.^19^ This oxygen requirement was then compared to oxygen production capacities calculated at three efficiency levels: 100%, 80% and 50%, categorised based on the operating capacity of hospital-based oxygen plants and private company’s oxygen plants.

Box 2 **Phased Prohibitory Orders and Surge Period Calculation in Nepal**

The surge period was considered based on the implementation of nationwide prohibitory order due to the delta variant. The Government of Nepal implemented this order in a phased manner for districts with over 500 active cases, commencing with the Kathmandu valley on 29 April 2021,^36^ and gradually expanding it across the entire country by 30 May 2023.^37^ The surge period was defined by adding the average hospitalisation duration between the first and last prohibitory order dates. An average hospitalisation period of 6 days was chosen based on the median length of time between symptom onset and hospitalisation, as studied by Faes C et al., which ranged from 3 to 10.4 days.^38^

These calculations were expressed in terms of J-size cylinders, a widely used unit in Nepal and internationally to facilitate comprehension and clarity. The J-size cylinder’s nominal content corresponds to 6800 L or 6.8 m^3^ of gaseous oxygen, with a water capacity of 47.2 L.^25^ The volume was standardised, assuming a filling pressure of 2000 psi (~137.9 bar), consistent with typical commercial medical oxygen supply. However, variations in compression and storage across sources may affect comparability and introduce estimation errors. Microsoft Excel spreadsheet served as the tool for collecting, analysing and subsequently presenting this data in a tabular format, complete with numerical values and corresponding percentages. Hospital-level aggregate data were collected with institutional cooperation; no patient-level data were used.

The study was approved by the Ethical Review Committee, Nepal Health Research Council (Reg. no. 442–2023).

### Patient and public involvement

Patients and/or the public were not involved in the design, conduct, reporting or dissemination plans of this research.

## Results

### COVID-19 hospitalisation trends

**Figure 1:**
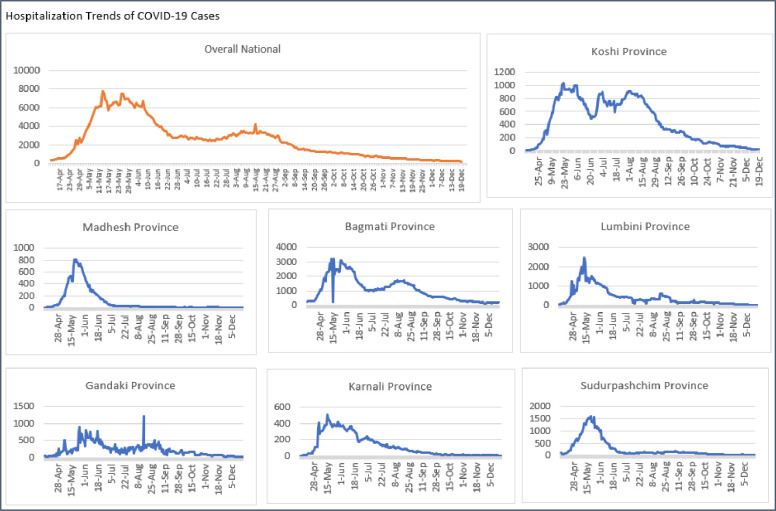
Trends of hospitalised COVID-19 cases spanning from 12 April 2021 to 19 December 2021, both on a national level as well as segmented by provinces.

This study captured the COVID-19 hospitalisation trends during the delta variant period (figure 1). On a national level, there was a gradual increase in the number of cases from the last week of April 2021 to the second week of June 2021. Similar trends were observed in the Madhesh, Bagmati, Lumbini, Karnali and Sudurpashcim provinces. However, Koshi and Gandaki provinces exhibited distinct patterns. In the case of Koshi province, cases increased from the first week of May 2021 to mid-August 2021, whereas non-symmetric patterns were evident for Gandaki province.

### Oxygen Production in Hospitals and Private Companies

Table 1 shows the medical oxygen production capacities of hospitals and private companies in Nepal. For example, out of 679 hospitals, only 7.4% (50 hospitals) had their own oxygen plants with a daily capacity of 3234 J-size cylinders. Additionally, the country had 30 oxygen plants operated by private companies, collectively capable of producing 18 670 J-size cylinders per day, with 85.2% of the oxygen output coming from these private manufacturers.

**Table 1:** Production Capacities of Oxygen in Hospitals and Private Companies

Province	Hospital production					Private manufacturer		% Oxygen Production Capacity of Private Manufacturer
	Number of hospitals*			Number of PSA plant	Oxygen production capacity (J-size Cylinder/day)	Number of private manufacturer	Oxygen production capacity (J-size Cylinder/day)	
	Public	Private	Total					
Koshi	31	74	105	8	508	4	2534	83.3
Madhesh	16	42	58	4	85	6	2996	97.2
Bagmati	63	219	282	19	1916	11	8700	82.0
Gandaki	19	52	71	6	202	2	1350	87.0
Lumbini	30	58	88	9	314	6	2970	90.4
Karnali	27	14	41	1	100	0	0	0.0
Sudurpaschim	15	19	34	3	109	1	120	52.4
Total	201	478	679	50	3234	30	18 670	85.2

* Source: Annual Report, Department of Health Services, 2077/78 (2020/21).

Across the provinces, a similar imbalance was noted, with private manufacturers contributing 82.0% to 97.2% of the oxygen output, except in the cases of Karnali and Sudurpaschim provinces. In Karnali, oxygen production was exclusively dependent on hospital facilities due to the absence of private company presence. Similarly, in Sudurpaschim, 52.4% of the oxygen output was sourced from a single private manufacturer’s plant, while the remaining supply originated from three hospital-based oxygen plants.

### Oxygen Requirement for COVID-19 Cases and Production Capacities

Online supplemental material table 2 presents data on the average daily hospitalisation COVID-19 patients, along with estimates for medical oxygen usage according to WHO standards and the respective production capacities across provinces from 6 May 2021 to 5 June 2021.

On average, the hospitals admitted 6323 patients daily, each requiring around 19,308 J-size oxygen cylinders per day. The oxygen production capacities, which were 21,904, 17,523 and 10,952 J-size cylinders at 100%, 80% and 50% efficiency levels, respectively. Notably, at 100% production efficiency, the production surpasses WHO’s estimation by 13.4%. However, at 80% and 50% production efficiency, the oxygen requirement only fulfils 90.8% and 56.7%, respectively.

Analysing the data by province, the highest patient admissions occurred in the Bagmati province, averaging 2544 patients daily, with a corresponding WHO oxygen requirement of 8,280 J-size cylinders. This province’s oxygen production, at 100% and 80% efficiency, exceeded the need by 28.2% and 2.6% respectively, while at 50% efficiency, it only met 64.1% of the requirement.

Conversely, Sudurpaschim province had the lowest COVID-19 patient count and the Karnali province exhibited the lowest oxygen production capacity, fulfilling only 9.4% of the oxygen requirement at 100% efficiency. The remaining provinces exhibited similar trends in COVID-19 patient numbers, WHO oxygen requirements and oxygen production capacities.

## Discussion

This study examined medical oxygen requirements and production capacities during the COVID-19 delta variant surge in Nepal. At full operational efficiency, the country’s oxygen production capacity met the requirement during the delta variant surge. However, given the fragility of Nepal’s infrastructure (eg,structural, electrical and geographic) and risks for natural disasters, maintaining round-the-clock production cannot be ensured at all times. Further, ongoing challenges include limited human resources during emergencies like pandemics, leading to workforce reductions due to personal and family issues.^26^ Equipment malfunctions or glitches can temporarily halt production,^27^ and Nepal’s unreliable power availability further complicates the situation.^5^

Findings revealed that at 80% production efficiency, only 90.8% of hospital requirements could be met. Similarly, at 50% efficiency, this drops to 56.7%. These numbers underscore significant production gaps, as produced oxygen is not only solely allocated to COVID-19 cases but also required for various medical conditions^28^ and surgeries.^29^ Furthermore, private sources also contribute to the supply chain, particularly for chronic patients who need oxygen for home use the and industrial sector. Thus, not all produced oxygen is exclusive for hospitals or COVID-19 patients.

In Nepal, oxygen production comes from both the hospitals and private manufacturers. However, 85.2% of the oxygen production comes from the private manufacturers. At this time, only 7.4% of healthcare facilities have their self-sustaining oxygen generation plants. This gap indicates a dependency on private company production for most healthcare facilities, particularly during periods of heightened demand. As such, private companies import liquid oxygen from India.^17^ But given the geographical proximity to India, there is risk of simultaneous disease outbreaks,^30^ which can potentially result in shared supply requirements for both nations during times of increased need.

A noticeable disparity of medical oxygen production and requirement emerges among provinces, particularly in the western provinces of Karnali and Sudurpaschim. Comparatively, these provinces exhibit notably lower oxygen production than the requirements. Some of the challenges include mountainous landscapes and rugged terrain, making it difficult for the delivery of medical supplies, goods and services.^31^ Further, there is limited transportation in these provinces, which compounds the challenges in transferring patients from rural areas who need higher levels of care where they can receive more life-saving interventions and medications. The health facilities in these provinces would greatly benefit from the installation of PSA plant and solar panels to ensure that the oxygen requirements are met.

### Policy Implication

The 2015 Constitution of Nepal grants provinces the authority to independently manage emergencies in their regions,^32^ with the option to request federal assistance if required. Regular assessment of each province’s capacities is crucial, particularly regarding oxygen production capacity. Meeting the expected provincial oxygen demand and continually evaluating hospital requirements should be placed as a high priority. As such, those hospitals with oxygen facilities should periodically assess their operations to identify gaps and requirements accurately.

Following WHO’s classification,^7^ Nepal included medical oxygen in its essential medicine list in 2021.^33^ To ensure its availability at healthcare facilities, a practical approach is to use PSA oxygen plants subject to a thorough preliminary needs and feasibility assessment. These compact systems can be conveniently transported and installed within hospitals, even though they require an upfront investment and regular maintenance expenses.^5^ PSA plants at hospitals present a sustainable and local solution for most of the healthcare facilities.^34^ Provincial and local governments can support the installation and maintenance of PSA plants through the allocation of budget. However, in the case of overall country-wise emergency preparedness and/or surge requirement, complementary use of all the three sources (liquid oxygen, ASU of private producers and hospital-based PSA plants) play a crucial role to meet the rising demand.

Optimisation of current capacity of oxygen production is critically important. This involves addressing crucial aspects, such as increasing the workforce to act as a backup in case of emergencies, improving storage facilities within various establishments or companies, maintaining equipment on a regular basis, alternate power solutions and tackling extra challenges to maintain a consistent and high-capacity production process without interruptions.^35^

### Limitation of the study

The study focuses on COVID-19 cases and recognises that hospitalisation rates and oxygen needs can vary depending on the variant and disease severity. Notably, the analysis did not include all COVID-19 variants. This implies the findings might not apply universally to every variant of the virus and disease severities.

The oxygen requirement calculation was based on hospitalised cases, overlooking instances where patients weren't admitted or reported. Some cases might not have been shared with HEOC by all hospitals. Additionally, not all severe cases were hospitalised due to factors like oxygen and bed shortages or patient lacking information about available medical services. During the delta wave of the pandemic, data on oxygen production or availability were collected through telephone interviews. This method may have introduced recall or reporting bias. Due to the prevailing conditions, independent verification using factory logs or hospital inventories was not feasible.

Moreover, oxygen concentrators were instrumental in managing oxygen requirement of patient with lower flow needs and were specifically a vital source of supplementary oxygen in Karnali and Sudurpaschim province. Available reports indicate limited deployment of oxygen concentrators in COVID-19 during the delta wave, particularly for severe cases requiring high flow rates. Although precise utilisation data were unavailable, local health authority guidance at the time prioritised centralised oxygen supplies. Likewise, liquid oxygen imported from India also aided the supply chain during the delta wave to some extent. These two sources are excluded from the study, given their relatively lower use for case management of patient infected with the delta variant of COVID-19. Further research is needed to understand the full requirement of medical oxygen consumption in Nepal.

Modelling production efficiency as 100%, 80% and 50% scenarios does not fully capture operational variability arising from equipment failures, maintenance lapses or workforce shortages. More detailed time-series data would be needed in future studies to refine these estimates. PSA plant performance and efficiency of commercial producers are also influenced by factors such as air quality, maintenance schedules and power supply stability, which can impact production reliability and should be considered in planning.

While J-size cylinders provide a standard unit for comparison, practical storage and distribution involve pipeline systems, transportation logistics and cylinder refilling times, all of which can affect real-world availability. These factors should be included in future modelling efforts to improve the accuracy and applicability of estimates.

## Conclusion

Medical oxygen is crucial for routine medical care and public health emergencies. To prepare for future emergencies, Nepal must assess its oxygen production capacities and needs using established frameworks. This assessment is vital for identifying gaps and creating effective strategies to handle sudden increases in oxygen demand.

Given the risk of reduced supply during high demand, hospitals should consider self-sufficiency measures. One effective approach is setting up individual oxygen plants within hospitals with an effective maintenance programme. This strategy strengthens a hospital’s ability to provide consistent oxygen during spikes in demand. At the same time, it is essential for the authorised body to closely monitor both the ASU sources and the liquid oxygen. Specific steps include prioritising investment in PSA plant maintenance, workforce training for plant operation, enforcing quality standards and establishing regulatory frameworks to monitor production capacity and distribution networks.

## Supplementary material

10.1136/bmjopen-2024-091189 online supplemental file 1

## Data Availability

Data are available upon reasonable request.
