# Supplementary material for: A cross-sectional quantitative analysis of production and requirements of medical oxygen during the COVID-19 pandemic in Nepal
Source: BMJ Open. 2025 Sep 11;15(9):e091189. doi: 10.1136/bmjopen-2024-091189 (PMC12519401; doi:10.1136/bmjopen-2024-091189)
Supplement: online supplemental file 1 [file bmjopen-15-9-s001.docx]

**Table 2. Average Daily Number of Hospitalized COVID-19 Patients, Medical Oxygen Estimation & Production Capacities by Provinces**

| **Provinces** | **Hospitalized COVID-19 patients** | | | **WHO Standard Cylinder Estimation (J sized cylinders** | | | **Oxygen Production Capacity (% fulfillment of requirement)** | | |
| --- | --- | --- | --- | --- | --- | --- | --- | --- | --- |
|  | **ICU** | **Wards /HDU** | **Total** | **ICU** | **Wards /HDU** | **Total** | **100% Efficiency (%)** | **80% Efficiency (%)** | **50% Efficiency (%)** |
| **Koshi** | 191 | 634 | 825 | 1212 | 1344 | 2556 | 3042 (119.0) | 2434 (95.2) | 1521 (59.5) |
| **Madhesh** | 149 | 403 | 552 | 947 | 854 | 1801 | 3081 (171.1) | 2465 (136.8) | 1541 (85.5) |
| **Bagmati** | 683 | 1861 | 2544 | 4339 | 3942 | 8280 | 10616 (128.2) | 8493 (102.6) | 5308 (64.1) |
| **Gandaki** | 122 | 253 | 375 | 775 | 536 | 1311 | 1552 (118.4) | 1242 (94.7) | 776 (59.2) |
| **Lumbini** | 146 | 1268 | 1415 | 930 | 2686 | 3616 | 3284 (90.8) | 2627 (72.7) | 1642 (45.4) |
| **Karnali** | 62 | 313 | 375 | 396 | 662 | 1058 | 100 (9.4) | 80 (7.6) | 50 (4.7) |
| **Sudurpaschim** | 44 | 193 | 236 | 277 | 408 | 685 | 229 (33.4) | 183 (33.4) | 115 (16.7) |
| **Total** | 1397 | 4926 | 6323 | 8877 | 10431 | 19308 | 21904 (113.4) | 17523 (90.8) | 10952 (56.7) |
