# Supplementary material for: A cross-sectional quantitative analysis of production and requirements of medical oxygen during the COVID-19 pandemic in Nepal
Source: BMJ Open. 2025 Sep 11;15(9):e091189. doi: 10.1136/bmjopen-2024-091189 (PMC12519401; doi:10.1136/bmjopen-2024-091189)
Supplement: Supplementary file 2 [file bmjopen-2024-091189.reviewer_comments.pdf]

PEER REVIEW HISTORY

BMJ Open publishes all reviews undertaken for accepted manuscripts. Reviewers are asked to complete a checklist review form and are provided with free text boxes to elaborate on their assessment. These free text comments are reproduced below.

ARTICLE DETAILS

Title (Provisional)

A cross-sectional quantitative analysis of production and requirements of medical oxygen during the COVID-19 pandemic in Nepal

Authors

Adhikari, Samir Kumar; Aryal, Yeshoda; Nepal, Anant; Bingham, Melissa Beth; Neupane, Subash; Basnet, Ashok; Singh, Amit Kumar; Prajapati, Bigyan; Sthapit, Deepesh; Devkota, Gaurav; Rana, Samriddha

---

VERSION 1 - REVIEW

**Reviewer**	**1**
**Name**	**Hosseinzadeh Hejazi, Sayed**
**Affiliation**	**Amirkabir University of Technology**
**Date**	**23-Feb-2025**
**COI**	**None**

Review of "A cross-sectional quantitative analysis of production and requirements of medical oxygen during the COVID-19 pandemic in Nepal"

This study presents a quantitative assessment of medical oxygen production and demand in Nepal during the COVID-19 pandemic, particularly the Delta wave. The research employs a cross-sectional methodology, utilizing secondary data from the Health Emergency Operation Centre. The study highlights discrepancies between oxygen supply and demand, identifies key challenges in infrastructure, and suggests policy measures to enhance oxygen availability in emergencies. Given the study’s relevance to public health and healthcare system resilience, its findings can inform national-level planning for future crises.

From an engineering perspective, the study effectively quantifies production capacities and efficiency scenarios (100%, 80%, and 50%). However, there are areas where a more rigorous technical approach to data analysis, modeling, and validation of assumptions could enhance its robustness.

Major Points

1. Assessment of Oxygen Production and Demand

o The study provides a valuable national and provincial-level breakdown of oxygen production, but its reliance on telephone surveys for data collection raises concerns about accuracy and potential bias. Incorporating independent validation (e.g., factory logs or hospital inventory records) would strengthen reliability.

o The assumption that hospital and private production efficiencies can be modeled as simple percentages (100%, 80%, and 50%) does not account for factors like operational variability, equipment failures, and workforce shortages, which could introduce significant uncertainties.

2. Methodology and Data Representation

o The use of J-size cylinders as the standard unit is reasonable for uniform comparison, but some additional context on real-world storage and distribution constraints (e.g., pipeline systems, cylinder refilling logistics) would improve the practical relevance of the findings.

o The study does not specify the pressure at which J-size cylinders are filled, which is critical for ensuring an accurate comparison between different oxygen sources. Oxygen production from PSA plants, liquid oxygen systems, and cylinder refilling stations operate at different pressures, and without this detail, it is unclear whether the reported production capacities are directly comparable. The authors should clarify the assumed pressure of J-size cylinders and ensure that all comparisons account for differences in gas compression and storage conditions.

o The study does not fully account for potential "hidden demand," such as patients who were unable to access hospitals due to capacity constraints. A sensitivity analysis incorporating different hospitalization scenarios could make the results more robust.

o The study briefly mentions that oxygen concentrators were used, particularly in Karnali and Sudurpaschim provinces, but excludes them from the analysis, stating that their use was relatively lower for managing COVID-19 cases during the Delta wave. However, no evidence or data is provided to support this claim. Given that oxygen concentrators are a key source of decentralized oxygen supply, especially in resource-limited settings, the authors should either provide quantitative evidence for their limited use or discuss their potential role in addressing oxygen shortages. Including this information would provide a more comprehensive picture of Nepal’s oxygen supply landscape.

3. Technical and Engineering Considerations

o The study discusses Pressure Swing Adsorption (PSA) oxygen plants but does not elaborate on key engineering constraints, such as air quality, maintenance schedules, and power supply stability, all of which can impact long-term production reliability.

4. Policy Implications and Recommendations

o The paper appropriately calls for decentralized oxygen production, yet it does not propose concrete steps for implementation. Additional details on infrastructure investment needs, training programs, and regulatory frameworks would improve the policy relevance.

o The reliance on imported liquid oxygen from India presents a significant risk, especially during regional crises. A comparative analysis of domestic production expansion versus import dependency would provide a stronger case for policy action.

Minor Points

• Terminology and Clarity: Some technical terms related to oxygen production could be better defined for a mixed medical and policy audience. For example, explaining how PSA compares to cryogenic separation in terms of efficiency and cost would add clarity.

• Figures and Tables: The tables provide useful data, but some could be improved by adding trends over time rather than just static snapshots of capacity versus demand.

• Grammar and Style: Minor grammatical errors exist, such as "However, if operational efficiency drops to 50%, the fulfillment rate diminished to 56.7%," which should be "However, if operational efficiency drops to 50%, the fulfillment rate diminishes to 56.7%."

• Ethical Considerations: The study mentions approval from Nepal’s Health Research Council, but a statement on whether individual hospitals consented to their data being used could be included for completeness.

Overall, this study provides valuable insights into Nepal’s medical oxygen supply chain challenges during COVID-19. The analysis is well-structured but could be strengthened with a more technical approach to efficiency modeling, sensitivity analyses, and engineering constraints. The policy recommendations are reasonable, though more concrete implementation strategies would improve their impact. Addressing these areas would enhance the study’s utility for both policymakers and engineering professionals involved in healthcare infrastructure.

**Reviewer**	**2**
**Name**	**Devereaux, Asha**
**Affiliation**	**California Emergency Medical Services Authority**
**Date**	**20-May-2025**
**COI**	**None**

The authors are to be commended on their comprehensive efforts to map oxygen supply in their region. Their efforts will hopefully serve as a basis for future disaster preparedness to meed the oxygen medical supply chain.

VERSION 1 - AUTHOR RESPONSE

SN	Peer Review Comments	Changes Made
This study presents a quantitative assessment of medical oxygen production and demand in Nepal during the COVID-19 pandemic, particularly the Delta wave. The research employs a cross-sectional methodology, utilizing secondary data from the Health Emergency Operation Centre. The study highlights discrepancies between oxygen supply and demand, identifies key challenges in infrastructure, and suggests policy measures to enhance oxygen availability in emergencies. Given the study's relevance to public health and healthcare system resilience, its findings can inform national-level planning for future crises.<br>From an engineering perspective, the study effectively quantifies production capacities and efficiency scenarios (100%, 80%, and 50%). However, there are areas where a more rigorous technical approach to data analysis, modeling, and validation of assumptions could enhance its robustness.		
Reviewer 1: Dr. Sayed Hosseinzadeh Hejazi, Amirkabir University of Technology: (Major Point)		
1.	Assessment of Oxygen Production and Demand:<br><br>• The study provides a valuable national and provincial-level breakdown of oxygen production, but its reliance on telephone surveys for data collection raises concerns about accuracy and potential bias. Incorporating independent validation (e.g., factory logs or hospital inventory records) would strengthen reliability.<br><br>• The assumption that hospital and private production efficiencies can be modeled as simple percentages (100%, 80%, and 50%) does not account for factors like operational variability, equipment failures, and workforce shortages, which could introduce significant uncertainties.	***4.2 Limitation of the Study (Page No 9)***<br>During the delta wave of the pandemic, data on oxygen production or availability were collected through telephone interview. This method may have introduced recall or reporting bias. Due to the prevailing conditions, independent verification using factory logs or hospital inventories was not feasible.<br><br>***4.2 Limitation of the Study (Page No 9)***<br>"We recognize that modeling production efficiency as 100%, 80%, and 50% scenarios does not capture operational variability from equipment failures, maintenance lapses, or workforce shortages. Future studies with detailed time-series data could refine these estimates."

SN	Peer Review Comments	Changes Made
2.	Methodology and Data Representation:<br><br>• The use of J-size cylinders as the standard unit is reasonable for uniform comparison, but some additional context on real-world storage and distribution constraints (e.g., pipeline systems, cylinder refilling logistics) would improve the practical relevance of the findings.<br><br>• The study does not specify the pressure at which J-size cylinders are filled, which is critical for ensuring an accurate comparison between different oxygen sources. Oxygen production from PSA plants, liquid oxygen systems, and cylinder refilling stations operate at different pressures, and without this detail, it is unclear whether the reported production capacities are directly comparable. The authors should clarify the assumed pressure of J-size cylinders and ensure that all comparisons account for differences in gas compression and storage conditions.<br><br>• ~~The study does not fully account for potential "hidden demand," such as patients who were unable to access hospitals due to capacity constraints. A sensitivity analysis incorporating different hospitalization scenarios~~	***4.2 Limitation of the Study (Page No 9 and 10)***<br>While J-size cylinders provide a standard unit for comparison, practical storage and distribution involve pipeline systems, transportation logistics, and cylinder refilling times, all of which can affect real-world availability. These factors should be included in future modeling efforts to improve the accuracy and applicability of estimates.<br><br>***2. Methods (Page No 4)***<br>The volume was standardized assuming a filling pressure of 2000 psi (~137.9 bar), consistent with typical commercial medical oxygen supply. However, variations in compression and storage across sources may affect comparability and introduce estimation errors.<br><br>***No Change in Manuscript:***<br>We agree this is a limitation. However, data on unmet hospitalization demand during the crisis were not available. We note this limitation but cannot conduct a sensitivity analysis without such data.<br><br>***4.2 Limitation of the Study (Page No 9)***<br>Available reports indicate limited deployment of oxygen concentrators in COVID-19 during the Delta wave, particularly for severe cases requiring high flow rates. Although precise utilization data were unavailable, local health authority guidance at the time prioritized centralized oxygen supplies.

SN	Peer Review Comments	Changes Made
	~~could make the results more robust.~~<br><br>• The study briefly mentions that oxygen concentrators were used, particularly in Karnali and Sudurpaschim provinces, but excludes them from the analysis, stating that their use was relatively lower for managing COVID-19 cases during the Delta wave. However, no evidence or data is provided to support this claim. Given that oxygen concentrators are a key source of decentralized oxygen supply, especially in resource-limited settings, the authors should either provide quantitative evidence for their limited use or discuss their potential role in addressing oxygen shortages. Including this information would provide a more comprehensive picture of Nepal's oxygen supply landscape.	
3.	Technical and Engineering Considerations:<br><br>• The study discusses Pressure Swing Adsorption (PSA) oxygen plants but does not elaborate on key engineering constraints, such as air quality, maintenance schedules, and power supply stability, all of which can impact long-term production reliability.	***4.2 Limitation of the Study (Page No 9)***<br>PSA plant performance and efficiency of commercial producers is also influenced by factors such as air quality, maintenance schedules, and power supply stability, which can impact production reliability and should be considered in planning.

SN	Peer Review Comments	Changes Made
4.	Policy Implications and Recommendations:<br><br>• The paper appropriately calls for decentralized oxygen production, yet it does not propose concrete steps for implementation. Additional details on infrastructure investment needs, training programs, and regulatory frameworks would improve the policy relevance.<br><br>• ~~The reliance on imported liquid oxygen from India presents a significant risk, especially during regional crises. A comparative analysis of domestic production expansion versus import dependency would provide a stronger case for policy action.~~	***5. Conclusion (Page No 10):***<br>Specific steps include prioritizing investment in PSA plant maintenance, workforce training for plant operation, enforcing quality standards, and establishing regulatory frameworks to monitor production capacity and distribution networks.<br><br>***No Change in Manuscript:***<br>"We appreciate the suggestion. Our study focused on quantifying supply-demand gaps during the crisis. A formal cost-benefit analysis of domestic capacity expansion vs. import reliance requires additional economic data and modeling beyond this paper's scope."
Reviewer 1: Dr. Sayed Hosseinzadeh Hejazi, Amirkabir University of Technology: (Minor Points)		
1.	Terminology and Clarity:<br><br>• Some technical terms related to oxygen production could be better defined for a mixed medical and policy audience. For example, explaining how PSA compares to cryogenic separation in terms of efficiency and cost would add clarity.	***1. Introduction (Page No 3):***<br>Although in smaller number, some hospitals in Nepal also operated in-house Pressure Swing Adsorption (PSA) oxygen plants. PSA systems separate oxygen from air using adsorption materials, typically achieving approximately 90-95% purity at moderate cost. In contrast, cryogenic separation involves liquefaction and distillation, enabling oxygen purity greater than 99%, but with higher capital and operational costs (18).<br><br>***6. References (Page No 13):***<br>18. World Health Organization. Foundations of medical oxygen system, 17 February 2023. Available From: https://www.who.int/publications/i/item/WHO-2019-nCoV-Clinical-Oxygen-2023.1
2.	Figures and Tables:	

<table>
<tr><th>SN</th><th>Peer Review Comments</th><th>Changes Made</th></tr>
<tr><td></td><td>• ~~The tables provide useful data, but some could be improved by adding trends over time rather than just static snapshots of capacity versus demand~~.</td><td>No Change in Manuscript:
“We agree that trends over time would strengthen the analysis. Unfortunately, consistent time-series data on production and demand were unavailable from HEOC records for the Delta wave period.”</td></tr>
<tr><td>3.</td><td>Grammar and Style:

• Minor grammatical errors exist, such as "However, if operational efficiency drops to 50%, the fulfillment rate diminished to 56.7%," which should be "However, if operational efficiency drops to 50%, the fulfillment rate diminishes to 56.7%."</td><td>Abstract (Page 1)
“However, if operational efficiency drops to 50%, the fulfillment rate diminishes to 56.7%.”</td></tr>
<tr><td>4.</td><td>Ethical Considerations:

• The study mentions approval from Nepal’s Health Research Council, but a statement on whether individual hospitals consented to their data being used could be included for completeness.</td><td>2. Methods (Page 4)
Hospital-level aggregate data were collected with institutional cooperation; no patient-level data was used.”</td></tr>
<tr><td colspan="3">Overall, this study provides valuable insights into Nepal’s medical oxygen supply chain challenges during COVID-19. The analysis is well-structured but could be strengthened with a more technical approach to efficiency modeling, sensitivity analyses, and engineering constraints. The policy recommendations are reasonable, though more concrete implementation strategies would improve their impact. Addressing these areas would enhance the study’s utility for both policymakers and engineering professionals involved in healthcare infrastructure.</td></tr>
<tr><td colspan="3">Reviewer 2: Dr. Asha Devereaux, California Emergency Medical Services Authority: The authors are to be commended on their comprehensive efforts to map oxygen supply in their region. Their efforts will hopefully serve as a basis for future disaster preparedness to need the oxygen medical supply chain.</td></tr>
</table>

VERSION 2 - REVIEW

**Reviewer**	**1**
**Name**	**Hosseinzadeh Hejazi, Sayed**
**Affiliation**	**Amirkabir University of Technology**
**Date**	**16-Aug-2025**
**COI**	

The authors have clearly addressed the limitations of their study and the paper is now ready for publication in my view.
